# Prenatal phenotype of *PNKP*-related primary microcephaly associated with variants affecting both the FHA and phosphatase domain

**DOI:** 10.1038/s41431-021-00982-y

**Published:** 2021-10-25

**Authors:** Sonja Neuser, Ilona Krey, Annemarie Schwan, Rami Abou Jamra, Tobias Bartolomaeus, Jan Döring, Steffen Syrbe, Margit Plassmann, Stefan Rohde, Christian Roth, Helga Rehder, Maximilian Radtke, Diana Le Duc, Susanna Schubert, Luis Bermúdez-Guzmán, Alejandro Leal, Katharina Schoner, Bernt Popp

**Affiliations:** 1 Institute of Human Genetics, University of Leipzig Medical Center, Leipzig, Germany; 2 MVZ Dr. Eberhard & Partner Dortmund, Dortmund, Germany; 3 Department of Pediatrics, Hospital for Children and Adolescents, Heidelberg University Hospital, Heidelberg, Germany; 4 Praxis für Pränatalmedizin, Dortmund, Germany; 5 Department of Radiology and Neuroradiology, Klinikum Dortmund, Dortmund, Germany; 6 Department for Pediatric Radiology, University of Leipzig Medical Center, Leipzig, Germany; 7 Institute of Medical Genetics, Medical University Vienna, Vienna, Austria; 8 Institute of Pathology, Department of Fetal Pathology, Philipps University Marburg, Marburg, Germany; 9 Section of Genetics and Biotechnology, School of Biology, University de Costa Rica, San José, Costa Rica

**Keywords:** Paediatric neurological disorders, RNA splicing, Genetic testing, Genetics research

## Abstract

Biallelic *PNKP* variants cause heterogeneous disorders ranging from neurodevelopmental disorder with microcephaly/seizures to adult-onset Charcot–Marie–Tooth disease. To date, only postnatal descriptions exist. We present the first prenatal diagnosis of *PNKP*-related primary microcephaly. Pathological examination of a male fetus in the 18th gestational week revealed micrencephaly with extracerebral malformations and thus presumed syndromic microcephaly. A recessive disorder was suspected because of previous pregnancy termination for similar abnormalities. Prenatal trio-exome sequencing identified compound heterozygosity for the *PNKP* variants c.498G>A, p.[(=),0?] and c.302C>T, p.(Pro101Leu). Segregation confirmed both variants in the sister fetus. Through RNA analyses, we characterized exon 4 skipping affecting the *PNKP* forkhead-associated (FHA) and phosphatase domains (p.Leu67_Lys166del) as the predominant effect of the paternal c.498G>A variant. We retrospectively investigated two unrelated individuals diagnosed with biallelic *PNKP*-variants to compare prenatal/postnatal phenotypes. Both carry the splice donor variant c.1029+2T>C *in* *trans* with a variant in the FHA domain (c.311T>C, p.(Leu104Pro); c.151G>C, p.(Val51Leu)). RNA-seq showed complex splicing for c.1029+2T>C and c.151G>C. Structural modeling revealed significant clustering of missense variants in the FHA domain with variants generating structural damage. Our clinical description extends the *PNKP*-continuum to the prenatal stage. Investigating possible *PNKP*-variant effects using RNA and structural modeling, we highlight the mutational complexity and exemplify a *PNKP*-variant characterization framework.

## Introduction

Microcephaly, or rather micrencephaly (abnormally small brain) in the narrow sense, is defined as an occipitofrontal circumference (OFC) below –2 SD of the mean for (gestational) age and sex and can occur in isolated form or in a syndromic context [1]. If detected prenatally, it is classified as primary microcephaly (PM) in contrast to secondary microcephaly developing after birth. Infections, traumata, ischemic events, exposure to teratogens, and genetic disorders are possible etiologies [1, 2]. As head growth depends on normal neuronal tissue proliferation, requiring continuous cell division, several genetic neurodevelopmental and neurodegenerative disorders are caused by variants affecting DNA repair genes, highlighting the importance of the pathways in neurogenesis [3].

The polynucleotide kinase 3’-phosphatase (PNKP) has a dual kinase/phosphatase function and is involved in the repair of both single- and double-strand DNA breaks [4, 5]. In 2010, biallelic pathogenic *PNKP* variants were reported to cause “microcephaly, seizures, and developmental delay” (MIM# 613402) [6]. Over time, three additional neurological and neurodevelopmental diseases have been associated with *PNKP* variants. These range from “childhood-onset ataxia with oculomotor apraxia type 4” (MIM# 616267) [7] to “developmental and epileptic encephalopathy, type 10” (MIM# 613402) [8] and “adult-onset Charcot–Marie–Tooth disease, type 2B2” (MIM# 605589) [9, 10].

Most pathogenic *PNKP* variants described so far are either truncating or located in the C-terminal kinase domain [11]. While genotype–phenotype correlations have been attempted and C-terminal variants have been implied to cause the milder adult-onset diseases, no clear relation could yet be established. Instead, it has even been postulated that the pathogenic variants observed present with rather mild mutational effects, due to survivorship bias, and more damaging variants would result in non-viability [11].

Here, we describe the first prenatal identification of biallelic *PNKP* variants affecting the region between the N-terminal forkhead-associated (FHA) domain and the phosphatase domain causative for severe early onset of PM. We provide detailed descriptions based on prenatal imaging and syndrome-oriented fetal autopsies of two affected sibling fetuses and compare the fetal phenotype with two individuals with *PNKP*-associated disorder and literature cases. In addition, we performed RNA analyses to characterize aberrant splicing of identified variants and used structural modeling to investigate missense variants.

## Materials and methods

### Genetic analyses and review of *PNKP* variants

P2 and both parents underwent trio-exome sequencing. In P3 and P4, clinical exome sequencing (CES) was performed. Segregation was confirmed through Sanger sequencing in all. Technical details and primer sequences are provided in Supplementary notes. All *PNKP* variants have been submitted to ClinVar (Supplementary File S3 [12] sheet “PNKP_variants”).

A PubMed search using the term “PNKP” identified 38 distinct *PNKP* variants from 21 publications (searched on December 15, 2020, details in Supplementary File S3 [12] sheet “PNKP_variants”). Variants were standardized to the reference transcript NM_007254.3 (GRCh37/hg19) using Mutalyzer 2.0.32 and annotated as described previously [13] (see Supplementary File S3 [12] sheet “PNKP_variants”). *PNKP* variants were classified following ACMG guidelines [14].

### Clinical data collection

We used a questionnaire for retrospective phenotypical analysis with clinical terms (standardized using HPO [15]) based on a review of clinical associations in *PNKP*-disorders [6–10]. The sheet was sent for evaluation to the pediatric neurologist or pathologist, respectively, and available clinical reports were added. Pre-/postnatal measurements were compared to published standards [16] or WHO child growth charts [17]. Comprehensive results are found in Supplementary File S2 [12] sheet “clinical” and Supplementary Fig. S1.

### Fetal autopsy and RNA extraction from fetal tissue

Fetal pathological examination of P1 and P2 was performed as previously described [18] (details in Supplementary notes). Cryopreserved native skeletal muscle tissue of P2 was processed with QIAshredder (Qiagen, Hilden, Germany) and RNA was extracted according to the manufacturer’s protocol (RNeasy Mini, Qiagen, Hilden, Germany).

### RNA analyses

In family 1, we performed RT-PCR as described previously [19] using PAXgene RNA in the parents and fetal skeletal muscle RNA derived cDNA. In family 3, we performed RNA-seq from PAXgene RNA using the TruSeq RNA Library Prep Kit v2 and paired-end sequencing. Bioinformatic workup included an established pipeline from our institute. In brief, reads were demultiplexed, adapters trimmed, and overrepresented sequences removed before we aligned the reads to the hg38 reference. Alignments were visualized and inspected for aberrant splicing as described previously [20]. We applied iREAD [21] to quantify observed intron retention events. Please see details in the Supplementary notes.

### Analysis of missense variant spectrum

Disease-associated missense analysis in the linear protein, clustering analysis in 3D and structural modeling of missense variants using the crystal structure 2BRF [22] was performed as described previously [11, 13, 19] and is detailed in the Supplementary notes.

## Results

### Prenatal phenotype in two sibling fetuses

In the third pregnancy of a healthy non-consanguineous couple, routine sonography at 13 weeks gestational age (GA) revealed microcephaly, abnormal skull shape, and microretrognathia in the male fetus (P2). Follow-up ultrasound (US) examinations at 15 and 16 weeks GA displayed progression of the anomalies. Amniocentesis for genetic testing (trio-ES) was performed at 16 weeks GA. Severe fetal anomalies and supposing genetic background caused the parents to decide for termination of pregnancy in week 18+3.

**Fig. 1:**
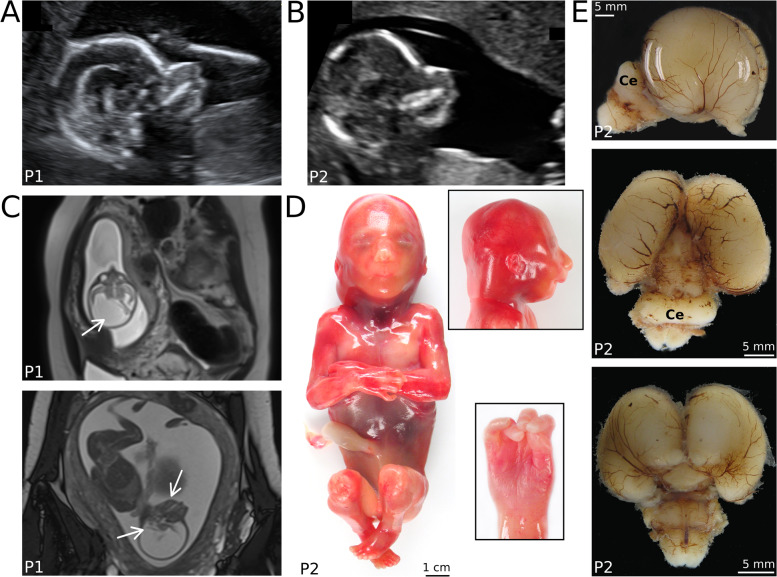
Prenatal clinical and autopsy results. **A** Prenatal ultrasonography of female fetus P1 at GA 19+1 weeks and **B** of male fetus P2 at GA 14+3 weeks show comparable micro-/brachycephaly with accentuated flattened short forehead and retromicrognathia in sagittal plane. **C** T2 axial MRI image at GA 20+6 weeks shows microcephaly and large supratentorial defects of the brain in occipital, parietal, and frontal regions with dilatation and fusion of both side ventricles. Lower panel: T2 TrueFISP sagittal image (GA 20+6 weeks) shows severe cerebellar hypoplasia as well as retrognathia. **D** Postmortem examination reveals the male fetus with appropriate development, arthrogryposis with camptodactyly (shown in detail), severe micro- and brachycephaly, short receding forehead, and retromicrognathia (shown in detail). **E** Brain in lateral, occipital, and frontal view presenting micrencephaly, especially affecting frontal lobes and moderate cerebellar hypoplasia (Ce).

A previous pregnancy had been terminated at 22 weeks GA after prenatal imaging had confirmed multiple anomalies in a female fetus (P1). US at 19+1 weeks GA had shown microcephaly, asymmetric skull shape, abnormal brain development, cerebellar hypoplasia, cataract of both eyes, and facial abnormalities. Prenatal MRI at 20+6 weeks GA confirmed microcephaly, large supratentorial defects of brain parenchyma in occipital, parietal and frontal regions, severe cerebellar hypoplasia, and dilatation/fusion of both lateral ventricles (Fig. 1C). Corpus callosum and septum pellucidum were not determinable. Bulbi of the eyes differed in size and signal.

Both fetuses were examined after TOP. The female fetus P1 (TOP at 22 weeks GA) measured 24.0 cm crown to heel length (standard: 26.2 ± 3.6 cm [16]) having a weight of 301 g (standard: 353 ± 125 g [16]). OFC was 15.0 cm (–4.3 SD; 5th percentile at GA: 17.3 cm [23]). Autopsy showed severe cerebral parenchymatous defects, profound hypoplasia of posterior cranial fossa, and confluence of the side ventricles. The findings were initially interpreted as arhinencephaly/holoprosencephaly. Extracerebral anomalies were not assessable (autolysis). Placenta appeared hypotrophic with signs of insufficiency.

The male brother fetus P2 (TOP at 18+3 weeks GA) was almost age-appropriate in terms of crown to heel length of 18.8 cm and weight 124.3 g (standard: 20 cm and 150 g), but had extremely small OFC (11.3 cm, –5.85 SD; 5th percentile at this GA: 14.1 cm [23]). Fetal autopsy confirmed severe micro-/brachycephaly, short receding forehead, narrow fontanelles, and associated facial dysmorphisms (hypertelorism, anteverted nares, long philtrum, small upper lip, small outer ears). Moreover, the fetus showed contractures according to early manifestation of arthrogryposis. Brain volume was reduced (about 40% of GA norm [16]) with slight enlargement of the ventricles. The frontal lobes were hypoplastic, occipital lobes were shortened and appeared wing-like. Temporo-parietal lobulation and corpus callosum were missing. Cerebellum was hypoplastic with a diameter of 1.2 cm (5th percentile for GA: 1.6 cm [23]). All examined brain sections appeared histologically normal. The findings were interpreted as micrencephaly without neuronal migration disorder or structural malformations. Discrete anisophthalmia/anisocoria with partial lens luxation due to dysplasia of the iris and persistent hyaloid artery of the left eye was noticed. There were no signs for external causes and no further organ abnormalities. Fetal autopsy results of P2 were suspected as monogenic syndromic type of microcephaly.

### Genetic analyses

Initial genetic investigations in P1, including conventional karyotyping, chromosomal microarray analysis, and a targeted holoprosencephaly sequencing panel were unremarkable. The recurrent pattern of PM in two successive pregnancies suggested a recessive syndromal type of PM. Thus, trio-ES was initiated after amniocentesis of P2. This analysis revealed the compound heterozygous *PNKP* variants c.498G>A, p.[(=),?] and c.302C>T, p.(Pro101Leu). The paternal variant c.498G>A is formally annotated as synonymous (p.(=)), but affects the last base of exon 4 and is predicted to disrupt the splice donor motif (p.(?)) with simple skipping of exon 4 resulting in an in-frame deletion between the FHA and the phosphatase domains. The maternal variant c.302C>T causes a proline to leucine missense change at the amino acid (AA) position 101 in the FHA domain. Both were initially classified as variants of unknown significance (VUS) according to ACMG recommendations (criteria: PM2, PP3). Subsequent segregation analysis in an archived amniotic fluid sample confirmed these two variants in compound heterozygous state in the affected fetus P1. Despite co-segregation evidence supporting pathogenicity, the variants’ classification remained VUS. Identified *PNKP* variants, variant effects, and classification are listed in Table 1. In P1 and P2, no other known pathogenic- or phenotypic-relevant variant was detected neither in *PNKP* nor in other disease-associated genes.

**Table 1:** Identified *PNKP* variants.

	P1 and P2	P1 and P2	P3	P3 and P4	P4
Chromosomal position	chr19:50368580G>A	chr19:50368384C>T	chr19:50368571A>G	chr19:50365626A>G	chr19:50370311C>G
c-code^a^	c.302C>T	c.498G>A	c.311T>C	c.1029+2T>C	c.151G>C
r-code		r.199_498del		r.[937_1029del,936_937ins936+1_937-1]	r.[151g>c,151_152ins151+1_152-1]
p-code^b^	p.(Pro101Leu)	p.Leu67_Lys166del	p.(Leu104Pro)	p.[Phe313_Pro343del, Leu312_Phe313ins*18]	p.[Val51Leu, Val51Argfs*68]
Effect	Missense variant	Splice region variant and synonymous variant	Missense variant	Splice donor variant and intron variant	Missense variant and splice region variant
InterVar	Uncertain significance	Uncertain significance	Uncertain significance	Pathogenic	Uncertain significance
ACMG	Likely pathogenic	Likely pathogenic	Uncertain significance	Likely pathogenic	Uncertain significance
Evidence	PS4_MOD; PM1_SUP; PM2_SUP; PM3; PP3	PVS1_STR; PM1_SUP; PM2_SUP	PM2_SUP; PM3; PP3	PVS1_STR; PM3_SUP; PS4_MOD; PM2_SUP	PM2_SUP; PM3; PP3

^a^Reference transcript: NM_007254.3.

^b^p-codes without round brackets are after RNA analyses.

### Postnatal phenotype in two unrelated individuals

The male index individual from family 2 (P3) was the first child of healthy non-consanguineous German parents with unremarkable family history. He was born at term after an uneventful pregnancy with a weight of 2915 g (–0.92 SD), a length of 52.0 cm (+1.12 SD) and OFC of 31.5 cm (–2.33 SD). Prenatal sonography was reported as unremarkable at GA 13 and 22 weeks. Postpartal, he showed muscular hypotonia and trigonocephaly caused by frontal synostosis. At the age of 5 months, he developed therapy refractory focal motor seizures with impaired awareness, bilateral tonic seizures, and multiple status epilepticus. Seizures were partially responsive to oxcarbazepine and valproic acid. Individual P3 was last reviewed at the age of 2 years and 4 months. His height was 83 cm (–2.04 SD) and weight was 8.4 kg (–4.49 SD). His OFC at the age of 2 years 6 months was 39 cm (–6.82 SD; Supplementary Fig. S1). Facial dysmorphism concerned typical stigmata of microcephaly. Neurological examination provided muscular hypotonia, ataxia, and oculomotor apraxia. He had moderate to severe global developmental delay with no speech and some limited passive understanding of words and signs but no active nonverbal communication. Developmental regression or behavioral abnormalities were not reported. Cranial MRI (cMRI) at the age of 5 months showed supra- and infratentorial white matter deficit, small corpus callosum, and a myelination delay. Brain imaging at the age of 2 years (Supplementary Fig. S2) showed extensive progressive microcephaly with simplified gyral pattern, an increasing white matter deficit, and the corpus callosum/cerebellum hypoplasia.

CES identified the two heterozygous *PNKP* variants c.1029+2T>C, p.(?) and c.311T>C, p.(Leu104Pro). Segregation analysis in the parents confirmed compound heterozygosity. The maternal variant c.1029+2T>C affects the canonical splice donor in intron 11, likely causing aberrant mRNA splicing. Skipping would result in an in-frame deletion of the 93 base pairs (bp) of the adjacent exon 11 causing a deletion of 31 AAs in the phosphatase domain. In fact, this variant was previously published, and RNA was analyzed using RT-PCR, which confirmed the predicted skipping effect [24]. The paternal base pair substitution c.311T>C causes a leucine to proline missense change at the AA position 104 in the FHA domain. The splice donor variant was initially classified as likely pathogenic while the missense variant was classified as VUS (criteria: PVS1, PM2 for c.1029+2T>C, p.(?); PM2, PM3 for c.311T>C, p.(Leu104Pro)).

The male index from family 3 (P4) was the first child of healthy non-consanguineous German parents with unremarkable family history. The pregnancy was complicated through a vanishing twin around GA 9 weeks, oligohydramnios, premature labor, and contractions at 17 and 24 weeks GA. Prenatal sonographic examination provided inconspicuous fetal development in gestational week 20. Individual P4 was born at term via caesarean section for arrest of labor with a weight of 3070 g (–0.58 SD), a length of 47 cm (–1.52 SD), and OFC of 32 cm (–1.94 SD). He presented with postnatal muscular hypotonia. At the age of 8 months, he showed motor development regression (loss of grabbing and turning). At age 12 months, he was able to speak first words and sit independently. At the last consultation at age 2 years and 9 months, his height was 86 cm (–2.32 SD), his weight was 11 kg (–2.36 SD), and his OFC was 42 cm (–5.14 SD), representing severe progressive microcephaly (Supplementary Fig. S1) and he had mild dysmorphic facial features (epicanthus, hypotelorism, and deep-set ears). Exploratory neurological examination showed muscular hypotonia and ataxia. He had mild global developmental delay. Brain imaging via cMRI and SPECT at the age of 2 years showed microcephaly without additional brain abnormalities (Supplementary Fig. S2).

CES revealed two heterozygous variants in *PNKP*, the heterozygous splice variant c.1029+2T>C, p.(?) and c.151G>C, p.(Val51Leu). Sanger sequencing confirmed the presence of both variants in P4 and their heterozygosity in the parents. The canonical splice variant was inherited from the mother. The paternal base pair substitution c.151G>C is annotated as missense change p.(Val51Leu) in the FHA domain but also affects the last nucleotide of exon 2, potentially affecting mRNA splicing. The splice donor variant was again classified as likely pathogenic while the missense variant was classified as VUS (criteria: PVS1, PM2 for c.1029+2T>C, p.(?); PM2, PM3 for c.151G>C, p.(Val51Leu)). In P3 and P4, no other known pathogenic- or phenotypic-relevant variant was detected neither in *PNKP* nor in other disease-associated genes.

### Analysis of missense variants in the FHA domain

Of 43 total unique variants reported here and in the literature (19 missense ~44.2% and two in frame AA deletions ~4.7%), seven missense variants (7/19, ~36.8%) are located in the FHA domain, four (4/19, ~21.1%) in the phosphatase and eight (8/19, ~42.1%) in the kinase domain. Mean values of the CADD scores regarding the FHA (22.2), phosphatase (23.5), and kinase (21.5) domains are significantly higher than for the linker domain (*p* < 2e–16, one-way ANOVA). These regions also contain all missense variants reported as (likely) pathogenic and disease-associated missense VUS. The linker stands out with a mean CADD score of 13.8 and the lack of disease-associated missense variants.

**Fig. 2:**
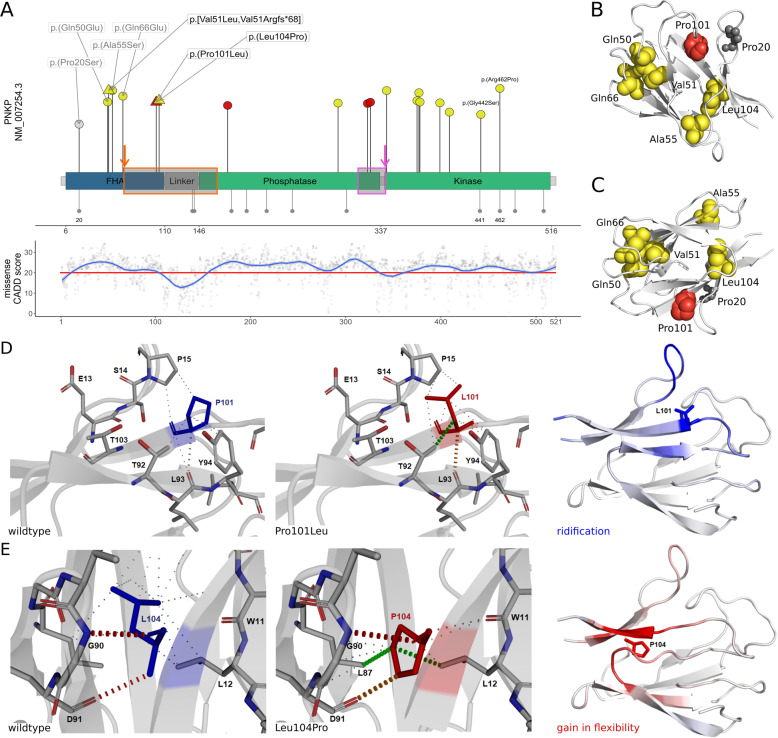
Missense variants. **A** Schematic depiction of the *PNKP* protein with FHA in blue, Phosphatase and kinase domain in green and linker region in gray (based on Uniprot Q96T60 and [20]). Disease-associated missense variants are displayed toward the top, red shapes show (likely) pathogenic variants, yellow shapes show variants of unknown significance, and gray shapes show (likely) benign variants. The variants identified in the four individuals reported here are depicted as triangles (black labels), variants from the literature as dots (gray labels). The length of the segments corresponds to each variant’s CADD score. Gray dots downwards show homozygous missense variants from gnomAD, the dot size represents the logarithm of the allele count. AA positions 20, 441, and 462 are labeled for orientation. In the panel below, a generalized additive model shows the CADD values for all possible missense variants (red horizontal line = recommended cut-off [20]). Additional boxes show the affected regions of the in-frame deletions resulting from the synonymous variant c.498G>A in orange or the splicing variant c.1029+2T>C in pink. **B**, **C** Two representative views of the FHA domain crystal structure (based on 2BRF). AAs of missense variants are depicted in gray for benign variants (Pro20), in yellow for VUS (Gln50, Val51, Ala55, Gln66, and Leu104), and in red for pathogenic variants (Pro101). All missense variants classified as unknown significance or (likely) pathogenic affect conserved residues in beta sheets. **D** The substitution Pro101Leu alters the local interatomic interaction of the protein, generating important steric clashes at the same time it decreases molecule stability. The most right panel represents the ΔVibrational Entropy Energy derived from the Pro101Leu variant, showing a local rigidification of the protein. **E** The Leu104Pro variant generates the opposite effect observed for Pro101Leu as it introduces a proline in the core of the FHA domain, destabilizing the protein domain. The most right panel shows the ΔVibrational Entropy Energy with a local gain in flexibility.

All three missense variants identified in P1–P4 are located in the FHA domain. Review of missense variants from the literature revealed four additional disease-associated variants in this domain (Fig. 2A). Beside the c.58C>T, p.(Pro20Ser) variant, which we classified as likely benign due to homozygous occurrence in reference populations (gnomAD), all missense variants previously reported as disease-associated in the FHA domain were classified as VUS using automated ACMG interpretation. Manual curation led to an evaluation as likely pathogenic for c.302C>T, p.(Pro101Leu). Three missense variants outside the FHA domain were also evaluated as likely pathogenic (c.526C>T, p.(Leu176Phe); c.968C>T, p.(Thr323Met); c.976G>A, p.(Glu326Lys)). ACMG [14] classifications of all variants can be found in Supplementary File S3 [12], sheet “PNKP_variants.”

The spatial distribution of AA residues affected by missense variants in the FHA domain (2BRF from RCSB Protein Data Bank) showed that most disease-associated missense variants affect conserved residues in beta sheets (Supplementary Fig. S3B). Analysis with mutation3D revealed clustering of the affected AA positions 50, 51, 55, 66, 101, and 104 in the FHA domain with a significant *p* value (bootstrapping) of 0.0112 (Fig. 2B, C).

According to Missense3D, the c.302C>T, p.(Pro101Leu) variant triggers a local steric clash alert (Fig. 2D). In addition, based on the DynaMut web server predictor, the effect of this variant is stabilizing and the ΔVibrational Entropy Energy between wildtype and mutant structure is predicted to slightly decrease molecule flexibility (Fig. 2D). The c.311T>C, p.(Leu104Pro) substitution introduces a buried proline in the core of the protein domain, which tends to be particularly damaging with its restricted backbone conformation (Fig. 2E). In fact, according to DynaMut, the variant has the opposite effect of p.(Pro101Leu) and is predicted to be destabilizing with increase of the molecule flexibility (Fig. 2E). Comprehensive results for variants in the FHA domain are listed in Table 2.

**Table 2:** Structural modeling results for missense variants in the FHA domain.

p-code^a^	p.(Gln50Glu)	p.(Val51Leu)	p.(Ala55Ser)	p.(Gln66Glu)	p.(Pro101Leu)	p.(Leu104Pro)
Case or reference	PMID: 31167812	P4^b^	PMID: 24965255	PMID: 30956058	P1 and P2	P3
CADD PHRED score v1.6	26.5	22.7	25.0	26.0	27.9	28.5
Missense3D	Replaces a buried uncharged residue with a charged residue	No structural damage	No structural damage	Replaces a buried uncharged residue with a charged residue	Local steric clash alert	Introduces a buried proline in the core of the protein domain
DynaMut	Destabilizing (ΔΔG: –0.362 kcal/mol) Increase of molecule flexibility (ΔΔSVib ENCoM: 0.357 kcal/mol*K)	Destabilizing (ΔΔG: –0.670 kcal/mol) Little decrease of molecule flexibility (ΔΔSVib ENCoM: –0.591 kcal/mol*K)	Stabilizing (ΔΔG: 0.989 kcal/mol) Little decrease of molecule flexibility (ΔΔSVib ENCoM: –0.345 kcal/mol*K).	Stabilizing (ΔΔG: 0.294 kcal/mol) Very little increase of molecule flexibility (ΔΔSVib ENCoM: 0.084 kcal/mol*K)	Stabilizing (ΔΔG: 1.510 kcal/mol) Slightly decrease of molecule flexibility (ΔΔSVib ENCoM: –0.287 kcal/mol*K)	Destabilizing (ΔΔG: –1.608 kcal/mol) Increase of molecule flexibility (ΔΔSVib ENCoM: 0.615 kcal/mol*K)

^a^Reference transcript: NM_007254.3.

^b^The c.151G>C base exchange observed in P4 causes a complex protein level (p.[Val51Leu,Val51Argfs*68]) p.[Val51Leu,Val51Argfs*68].

### RNA analyses

**Fig. 3:**
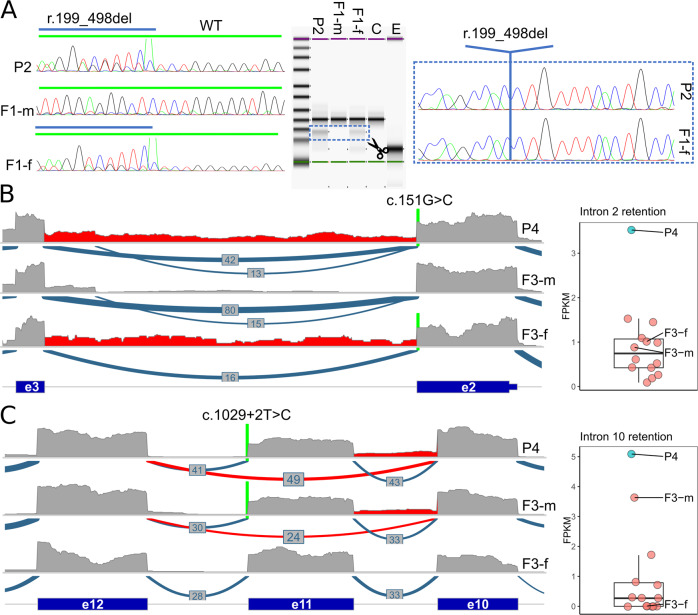
RNA analyses. **A** RT-PCR and subsequent Sanger sequencing (left panel) on cDNA derived from RNA of individual P2 and both parents (F1-m, F1-f) to determine the effect of the c.498G>A variant. In P2 and the heterozygous carrier father a smaller product is present (middle panel). Right panel: skipping of exon 4 (r.199_498del) causes an in-frame deletion on protein level (p.Leu67_Lys166del). WT wildtype, C control, E empty. **B** Coverage and Sashimi-plot (left panel) of RNA-seq from family 3 for the exon 2–exon 3 region. The c.151G>C variant is indicated with a green line. Coverage plot for P4 and the heterozygous carrier father (F3-f) (highlighted in red), indicating intron 2 retention (r.151_152ins151+1_152-1). This is confirmed by the FPKM values shown as box and scatter plots (right side), where P4 is the only outlier. **C** Coverage and Sashimi-plot (left panel) of RNA-seq data from family 3 for the exon 10–exon 12 region. The position of the c.1029+2T>C variant is indicated with a green line. The Sashimi-plot shows skipping of exon 11 (r.937_1029del; highlighted in red) in P4 and the heterozygous carrier mother (F3-m), which is predicted to cause an in-frame deletion (p.Phe313_Pro343del). In addition, the coverage plot indicates retention of intron 10 (r.936_937ins936+1_937-1; red highlight) in P4 and F3-m, which is confirmed by the FPKM values for this exon (right panel and predicted to cause a truncated protein (p.Leu312_Phe313ins*18).

To determine the effect of the paternally inherited c.498G>A variant in P1 and P2, we performed RT-PCR and Sanger sequencing on cDNA from RNA of individual P2 and both parents (F1-m, F1-f). In addition to the wildtype product, we identified a second, smaller PCR product in the sample of the father and P2. Sanger sequencing of this smaller product revealed skipping of the 300 base pair long exon 4 (r.199_498del) leading to an in-frame deletion of the 100 AAs of exon 4 on protein level (p.Leu67_Lys166del) (Fig. 3A).

To elucidate the effect of the paternal base pair substitution c.151G>C, affecting the last base of exon 2, and proof the recently published aberrant splicing effect [24] of the maternally inherited canonical donor variant c.1029+2T>C, we performed RNA sequencing from PAXgene blood sample of P4 and both parents (Fig. 3). RNA sequencing data in the sample of P4 and the heterozygous carrier father (F3-f) showed mainly normal splicing events for the exon 2/3 region with nucleotide exchange c.151G>C leading to the missense change p.Val51Leu one consequence from this allele. The sequencing reads also support an aberrant transcript with retention of intron 2 (r.151_152ins151+1_152-1), which is predicted to cause a frameshift and a premature stop codon (p.Val51Argfs*68). Overall the c.151G>C base exchange causes a complex effect on RNA and protein level (p.[Val51Leu,Val51Argfs*68]) (Fig. 3B). Estimating the predominant aberrant transcript and effect is complicated by the presence of the second variant in the index and apparently incomplete nonsense mediated RNA-decay. Based on the allele fraction of the RNA-seq reads with the c.151G>C change in the father (10/35 overall), the two consequences from this allele (4 correctly spliced with base exchange, 6 with retention) are about equally expressed.

For the variant c.1029+2T>C inherited from the mother (F3-m) our RNA-seq data confirmed the previously described [24] and expected effect of exon 11 skipping (r.937_1029del) leading to an in-frame deletion of the 31 AAs (p.Phe313_Pro343del). We identified evidence for an intron 10 retention (r.936_937ins936+1_937-1) as a second aberrant transcript, which is predicted to cause a truncated protein (p.v) (Fig. 3C). Thus, also this canonical splice variant causes a complex mixture of aberrant transcripts (p.[Phe313_Pro343del,Leu312_Phe313ins*18]). Estimating the predominant effect is again complicated by multiple novel transcripts from both alleles in the index. From the spliced reads observed over the skipped exon in the mother (compare Fig. 3C), one would calculate an exon-inclusion ratio (or percent spliced in) of 56.8% (((30 + 33)/2)/((30 + 33)/2 + 24)) for exon 11. To restore a model with equal expression of the transcripts from both alleles in the mother and assuming no nonsense mediated decay, 7.5 transcripts would have to be assigned to the retention event (add 7.5 to the denominator) which would constitute ~11.9% (7.5/63) of all transcripts. This calculation is in agreement with the observed coverage profile between exon 10 and intron 10 (11/69, ~15.9%). Overall, this confirms that the predominant aberrant splice effect caused by the c.1029+2T>C variant is in-frame exon skipping.

## Discussion

Before our report, *PNKP* was already associated with a wide phenotypic spectrum, which could be related to its multidomain architecture. The FHA domain recruits PNKP to DNA damage sites [25], where it is involved in repair of both single- and double-strand breaks through its enzymatically active kinase and phosphatase domains [26, 27].

Despite the various *PNKP*-associated phenotypes [5–9, 28–30], prenatal presentations in humans, especially noticeable brain anomalies, were unreported to date. Our compilation of prenatal diagnostic procedures and fetal pathological examination of P1 and P2 revealed neurodevelopmental and neurodegenerative brain alterations comparable to those described in mouse models with neuronal tissue-specific inactivation of *PNKP* [31]. These include general hypoplasia of different cerebral and cerebellar regions, without a histologically recognizable neuronal migration disorder. Furthermore, the two fetuses showed a convincing phenotypic accordance with the previously described MCSZ phenotype [6]. While the exact pathomechanism of *PNKP-*associated microcephaly is still disputed, the prenatal manifestation represents the most severe outcome. This may arise from extreme genome instability in neurons with impaired development on the one hand and increased cell death on the other hand (e.g., extensive white matter deficit in P1).

With suspicion of recurrent microcephaly, fetal autopsy of P2 was oriented toward an underlying syndromic disorder. Missing macroscopic signs for holoprosencephaly such as hypotelorism, hypoplastic anterior cranial fossae, or absent cribriform plate of ethmoid bone in the first pathological examination (limited on microscopic level due to autolysis) together with the unremarkable holoprosencephaly panel analysis provide sufficient arguments to retrospectively rule out holoprosencephaly in P1. The synergy of unbiased exome sequencing for prenatal anomalies and exact phenotyping in a syndrome-oriented fetal autopsy in P2 highlight their importance for diagnosing an unusual manifestation of a known disease and evaluation of a novel variant of uncertain clinical significance.

Given the OFC values observed immediately after birth in P3 and P4, the presence of prenatal microcephaly in these individuals is obvious. The diagnosis of PM relies on US measurements in comparison to distributions for the respective GA and exclusion of exogenous causes such as infectious diseases. Until the recent Zika virus outbreak, no international standards and guidelines had been defined and research on the diagnostic performance of US measurements for fetal microcephaly was hampered by the overall rarity of the condition [32]. Performance for prenatal US diagnosis seems good at the more extreme ends (<–4 SD) or when additional brain anomalies are present [32], like in the case of the two fetuses of family 1. In addition, improved US technology and specialist training during the last years might have enabled these prenatal diagnoses.

**Fig. 4:**
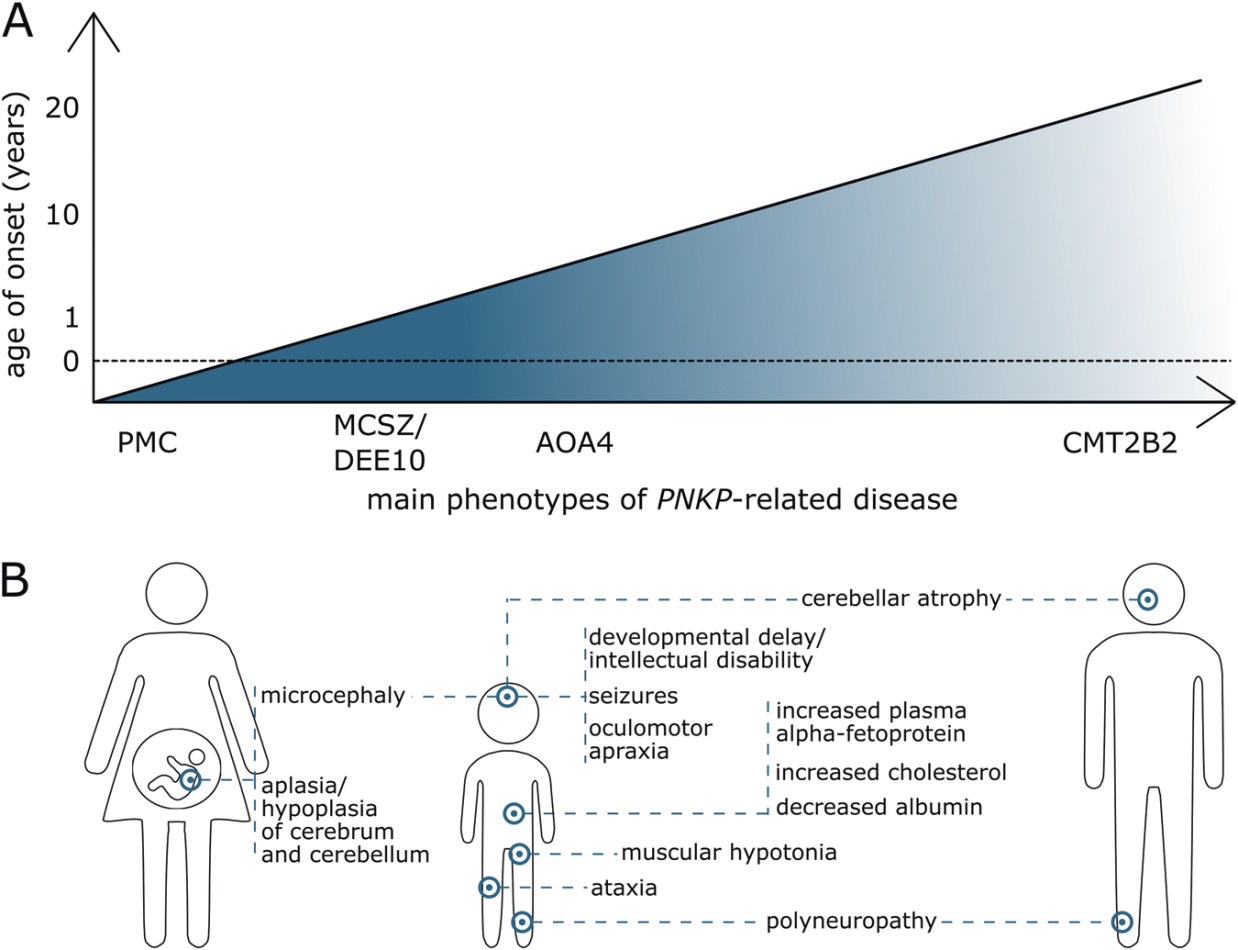
Phenotypes. **A** Schematic indicating the proposed phenotypic continuum in *PNKP*-related disease: MCSZ (microcephaly, seizure, and developmental delay; MIM# 613402) and developmental and epileptic encephalopathy (DEE10; MIM# 613402), AOA4 (ataxia-oculomotor apraxia type 4; MIM# 616267), and Charcot–Marie–Tooth disease, type 2B2 (CMT2B2; MIM# 605589). Prenatal manifestation is added as prenatal microcephaly (PMC). Interpreted severity of the disease is shown by color course. **B** Comparison of the main clinical aspects between prenatal period (left), childhood (middle), and adulthood (right). Aplasia/hypoplasia of the cerebrum (HP:0007364) and the cerebellum (HP:0007360) is exclusively described in the prenatal period. Microcephaly (HP:0000252) is one of the main features of the prenatal and childhood phenotype MCSZ. MCSZ and DEE10 also comprise developmental delay/intellectual disability (HP:0001263; HP:0001249), seizures (HP:0001250), and muscular hypotonia (HP:0001252). Ataxia (HP:0001251) and oculomotor apraxia (HP:0000657) are characteristic for AOA4. Distinct biochemical features (elevated alpha-fetoprotein HP:0006254, hypoalbuminemia HP:0003073, hypercholesterolemia HP:0003124) were mostly described in cases with AOA4. Overlap between childhood and adulthood presentation are cerebellar atrophy (HP:0001272) and polyneuropathy (HP:0000763).

We recommend interpreting the phenotypic presentations associated with biallelic variants in *PNKP* as continuous spectrum instead of the separated clinical entities MCSZ, AOA4, and CMT2B2, in agreement with previous suggestions [33]. In this disease model, our report adds the most severe, prenatal presentation with extreme microcephaly, and possibly unviable additional cerebral anomalies in the two sibling fetuses. With regard to the grade of severity, this is followed by postnatal microcephaly with severe to mild intellectual disability combined with further neurological symptoms like epilepsy and finally by adult-onset polyneuropathy as the mildest presentation (Fig. 4). Observing this clinical variability, the question arises whether there could be a relation to genetic variant context.

On a genomic level, *PNKP* stands out given the specific composition with many small introns toward the 3’ end when compared to the average human intron size [34]. Smaller introns are associated with a higher likelihood of intron retention [35], a mechanism we proved for two novel variants here. Except the RT-PCR analysis of the c.1029+2T>C variant, detailed RNA analyses of disease-associated *PNKP* variants were previously not reported, despite many intronic and splice sites affecting changes described [6, 24, 25, 28, 33]. The results of our RNA splicing analyses for one novel silent variant, one known splice donor variant and moreover one variant annotated as missense point toward a likely underappreciated pathomechanism. Predictions by computational tools implicate a splice effect for 15 of 43 (35%) *PNKP* variants, which implies a need for further functional evaluation. We show that complex splice events in *PNKP* can readily be assessed using RNA from peripheral blood. Expanding these analyses to newly identified variants will support variant pathogenicity interpretation and improve understanding of aberrant splicing for genes with similar exon configuration.

Next to the often complex and possibly hidden splice effects identified here, true AA substitutions are to date the most difficult variants to interpret. Evaluation of missense variants can be composed of evolutionary conservation, functional predictions, and genetic context as it is used comprehensively, e.g., in the CADD score [36]. Here we complemented the analysis of such scores in the linear protein model with structural protein modeling. Four variants identified in the three families affect the FHA domain. The AA residues affected by the missense variants p.(Gln50Glu), p.[Val51Leu,Val51Argfs*68], p.(Ala55Ser), p.(Gln66Glu), p.(Pro101Leu), and p.(Leu104Pro) show significant clustering with other AA changes described in the literature. Interestingly, the compound heterozygous variants identified in family 1 alter the same region of PNKP: the variant p.Leu67_Lys166del predominantly results in a deletion affecting parts of the FHA, linker, and phosphatase domain and thereby encompasses a total of around 20% of PNKP, while the second is the missense variant p.(Pro101Leu) located at the C-terminus of the FHA domain. Previously, no disease-associated variants were described in this particular PNKP region, which shows high conservation and absence of homozygous variants in population databases. The severity of the observed prenatal phenotype may either due to the combined interdomain effect of the large in frame deletion or serves as an intriguing confirmation of a “Wald’s domain” at the crossing between the FHA and linker domains, which has recently been proposed as a survivorship bias for disease-associated *PNKP* variants [11].

Taking into account the different mutational mechanisms of *PNKP* variants and the wide phenotypic spectrum, currently no clear genotype–phenotype correlation and therefore no accurate phenotypic prediction in association with a specific variant seems possible. While *PNKP* may be an extreme example, this is a common challenge in disorders with autosomal-recessive inheritance. In homozygous state, either because of consanguinity or due to founder variants, other effects of the haplotype can influence phenotype, while in compound heterozygous state the combination of two variants with possibly different effects each complicates accurate phenotype association. The latter is exemplified by the difference in clinical severity in individuals P3 and P4 who both carry the same splice variant c.1029+2T>C causing a mixture of protein effects (p.[Phe313_Pro343del, Leu312_Phe313ins*18]) on one allele and a different missense variant each on the second allele. Individual P4, who has the milder phenotype without seizures, carriers the splice region variant c.151G>C causing mixture of missense (p.Val51Leu) and truncating effect (p.Val51Argfs*68). In contrast, individual P3 who has severe neurodevelopmental delay, seizures, and extreme microcephaly (<–6 SD) carries the missense variant c.311T>C, p.(Leu104Pro), which lies in the same FHA region and close proximity of the variants in family 1. While this anecdotal correlation seems convincing, dysfunction of the phosphatase domain was already associated with *PNKP*-associated neurodevelopmental phenotype [11, 25], which we can support with variants in all three families affecting this domain. Systematic functional studies matched with standardized clinical assessment (HPO; see Fig. 4) will be needed to reach precise genotype–phenotype prediction.

Here, we extended the “*PNKP*-associated disorder continuum” to the prenatal period, complemented missense variant interpretation with 3D structure analysis and presented the first RNA-seq data used to elucidate exact effects of *PNKP* variants. The knowledge of distinct fetal phenotypes will be helpful for genetic variant assessment, especially those with unknown significance. Only with knowledge of variant pathogenicity and expected symptoms, we will be able to improve counseling in the prenatal setting, management in the postnatal period, prenatal diagnosis in subsequent pregnancies, and finally enable potential future treatments.

## Web resources

gnomAD browser: http://gnomad.broadinstitute.org/,

Mutalyzer: https://mutalyzer.nl,

ClinVar: https://www.ncbi.nlm.nih.gov/clinvar/,

Missense3D: http://missense3d.bc.ic.ac.uk/missense3d/,

Dynamut: http://biosig.unimelb.edu.au/dynamut/,

Mutation3D: http://mutation3d.org/.

## Supplementary information

File S1

## Data Availability

All data generated or analyzed during this study can be found either in the online version of this article at the publisher’s website or has been uploaded to Zenodo.
